# Short tandem repeat number estimation from paired-end reads for multiple individuals by considering coalescent tree

**DOI:** 10.1186/s12864-016-2821-0

**Published:** 2016-08-31

**Authors:** Kaname Kojima, Yosuke Kawai, Naoki Nariai, Takahiro Mimori, Takanori Hasegawa, Masao Nagasaki

**Affiliations:** 1 Tohoku Medical Megabank Organization, Tohoku University, 2-1, Seiryo-machi, Aoba-ku, Sendai, 980-8573 Japan; 2 Institute for Genomic Medicine, University of California, San Diego, 9500 Gilman Drive #0761, San Diego, 92093-0761 USA

**Keywords:** High-throughput sequencing, Short tandem repeat, Coalescent theory

## Abstract

**Background:**

Two types of approaches are mainly considered for the repeat number estimation in short tandem repeat (STR) regions from high-throughput sequencing data: approaches directly counting repeat patterns included in sequence reads spanning the region and approaches based on detecting the difference between the insert size inferred from aligned paired-end reads and the actual insert size. Although the accuracy of repeat numbers estimated with the former approaches is high, the size of target STR regions is limited to the length of sequence reads. On the other hand, the latter approaches can handle STR regions longer than the length of sequence reads. However, repeat numbers estimated with the latter approaches is less accurate than those with the former approaches.

**Results:**

We proposed a new statistical model named coalescentSTR that estimates repeat numbers from paired-end read distances for multiple individuals simultaneously by connecting the read generative model for each individual with their genealogy. In the model, the genealogy is represented by handling coalescent trees as hidden variables, and the summation of the hidden variables is taken on coalescent trees sampled based on phased genotypes located around a target STR region with Markov chain Monte Carlo. In the sampled coalescent trees, repeat number information from insert size data is propagated, and more accurate estimation of repeat numbers is expected for STR regions longer than the length of sequence reads.

For finding the repeat numbers maximizing the likelihood of the model on the estimation of repeat numbers, we proposed a state-of-the-art belief propagation algorithm on sampled coalescent trees.

**Conclusions:**

We verified the effectiveness of the proposed approach from the comparison with existing methods by using simulation datasets and real whole genome and whole exome data for HapMap individuals analyzed in the 1000 Genomes Project.

## Background

The progress of high-throughput sequencing (HTS) technologies enables the variant detection of each individual in genome-wide scale in practical time and with reasonable cost. From HTS data, various types of single nucleotide variant (SNV) calling methods have been proposed [1–4], and SNVs for more than a thousand of individuals were accurately detected [5]. However, unlike SNVs, we still have difficulty in accurately detecting structural variations such as genome insertions, genome deletions, short tandem repeat (STR) number polymorphisms, and copy number variations, especially from low coverage HTS data [6].

Some repeat number polymorphisms are associated with various disease phenotypes such as CAG repeats in the Huntingtin gene with Huntington’s disease [7]. From HTS data, several approaches such as lobSTR and RepeatSeq [8, 9] have been proposed for the estimation of repeat numbers in STR regions by directly counting repeat patterns in sequence reads spanning the regions. In these approaches, the accuracy on both the detection of STR variants and estimated repeat numbers is high. Another strategy is to use paired-end reads aligned to the flanking regions of the target STR region in the reference genome [10]. Insert size inferred from the aligned paired-end reads is longer than its actual size if the repeat number is smaller than that in the reference genome. On the other hand, the inferred insert size is shorter if the repeat number is larger. By detecting the difference between the inferred and actual insert size, repeat numbers are estimated. Since insert size is generally longer than sequence reads, this strategy can be used for estimating repeat numbers for relatively long STR regions that cannot be handled by the strategy counting repeat patterns in sequence reads. However, repeat numbers estimated from insert size data are less accurate than those from the strategy counting repeat patterns directly in the sequence reads, especially for low coverage HTS data.

We proposed a new statistical model named coalescentSTR that estimates repeat numbers for multiple individuals simultaneously from paired-end read distances by connecting the read generative model for each individual with their genealogy. In the model, the genealogy is represented with coalescent trees, which describe the ancestral history of multiple individuals on a local genome region backwards in time [11–13]. By considering the change in repeat numbers in coalescent trees in a natural manner, more accurate estimation of repeat numbers is expected. For the estimation of repeat numbers in the model, coalescent trees handled as hidden variables are sampled with Markov chain Monte Carlo (MCMC) according to phased genotypes around a target STR region. We proposed a new belief propagation method that calculates the loopy belief propagation [14] and the mixed-product belief propagation [15] by taking the summation on the sampled coalescent trees. By using the proposed belief propagation, approximated maximum configuration of repeat numbers in the model are searched for the estimation of repeat numbers.

In a simulation study, we used synthetically generated HTS data for STR regions mostly longer than read length, and showed the effectiveness of our model from the comparison with other existing methods, especially in handling more individuals. The effectiveness of our approach is also verified from the analysis of real whole exome data of HapMap JPT individuals and whole genome sequencing (WGS) data of HapMap CEU and GBR individuals analyzed in the 1000 Genomes Project (1KGP).

## Method

We describe a model considering insert size of paired-end reads for one individual and its extension to consider multiple individuals based on their unobserved genealogy. Procedures for the repeat number estimation are then explained.

### Repeat number estimation from paired-end read distance

We consider a statistical model that estimates repeat number in an STR region from paired-end read distance for one individual. We hereafter call the model a basic model. Let *s*^(*d*)^ be the start position of the forward read of the *d*th aligned read pair. We also let *e*^(*d*)^ be the end position of the reverse read of the *d*th aligned read pair. The insert size of the *d*th read pair or the length of the DNA fragment from which the read pair was generated is given by *e*^(*d*)^−*s*^(*d*)^, and we denote the insert size *e*^(*d*)^−*s*^(*d*)^ as *l*^(*d*)^.

If an *x* bp insertion variant exists between *s*^(*d*)^ and *e*^(*d*)^ in the genome of an individual, *l*^(*d*)^ is *x* bp shorter than the actual insert size of the *d*th read pair. On the other hand, if an *x* bp deletion variant exists between *s*^(*d*)^ and *e*^(*d*)^, *l*^(*d*)^ is *x* bp longer than its actual insert size. By detecting the difference between *l*^(*d*)^ and the actual insert size, the basic model estimates repeat numbers. Let *u*, *n*_*r*_, *n*_1_, and *n*_2_ be the length of a repeat pattern, the repeat number in reference genome, the repeat number on haplotype 1, and the repeat number on haplotype 2, respectively. If the DNA fragment for the *d*th read pair spans the STR region in haplotype 1, its actual insert size is given by *l*^(*d*)^+*u*·(*n*_1_−*n*_*r*_), and hence the probability of *l*^(*d*)^ is given by $\mathcal {F}(l^{(d)} + u \cdot (n_{1} - n_{r}))$, where $\mathcal {F}$ is the insert size distribution of the sequence data. If the start or end position of the DNA fragment is in the inside of the STR region, the DNA fragment cannot be used for estimating repeat numbers. Thus, *l*^(*d*)^ must be longer than *e*_*m*_−*s*^(*d*)^, where *e*_*m*_ is the end position of the STR region in the reference genome. We also exclude the DNA fragment longer than *K*, i.e., $\mathcal {F}(l)$ takes 0 for *l*>*K*, where *K* is a sufficiently large number and is set to 2,000 bp in our study. The generative probability of *l*^(*d*)^ is represented by normalizing $\mathcal {F}$ as follows:

$${}P(l^{(d)} \! \mid n)\! =\! \left\{\! \! \begin{array}{cl} \frac{\mathcal{F}(l^{(d)} + u \cdot (n - n_{r}))}{N(s^{(d)},n)} & \! \text{if \! \(l^{(d)}\! >\! e_{m} \! -\! s^{(d)}\! \) \&\! \(l^{(d)} \! \le\! K\! -\! u\! \cdot \! (n\! -\! n_{r})\)} \\ 0 & \text{otherwise} \end{array} \right.\! \!\!, $$

where *N*(*s*,*n*) is the normalization factor given by

$$N(s,n) = \sum\limits_{l=e_{m} - s + 1}^{K-u \cdot (n-n_{r})} \mathcal{F}(l + u \cdot (n-n_{r})). $$

Since each read pair is generated from one of two DNA sequences in equal probability, the likelihood of *l*^(*d*)^ is represented by

1 $$\begin{array}{@{}rcl@{}} {}\prod\limits_{d=1}^{D} P(l^{(d)} \! \mid\! n_{1}, n_{2}) = \prod\limits_{d=1}^{D} \frac{1}{2} \left(P(l^{(d)} \! \mid\! n_{1})\! + \! P(l^{(d)} \mid n_{2})\right), \end{array} $$

where *D* is the number of read pairs. We consider the maximum and minimum repeat numbers $n_{\max }$ and $n_{\min }$ and search the pair of *n*_*i*1_ and *n*_*i*2_ in $\{n_{\min }, \dots, n_{\max }\} \times \{n_{\min }, \dots, n_{\max }\}$ maximizing Eq. (1), which requires $O((n_{\max }-n_{\min }+1)^{2})$ time. The computational time in the basic model is mainly taken by the calculation of the normalization factor *N*(*s*,*n*), which requires $O(D(n_{\max }-n_{\min }+1)K)$ time in a naïve way. Thus, we propose an algorithm that calculates *N*(*s*,*n*) more efficiently by considering the following two recurrence formulae of *N*(*s*,*n*):

$$\begin{array}{@{}rcl@{}} N(s+1,n) &=& N(s,n) + \mathcal{F}(e_{m} - s + u \cdot (n-n_{r}))  \\ N(s,n+1) &=& N(s,n) - \sum\limits_{l=e_{m} - s + 1}^{e_{m} - s + u} \mathcal{F}(l + u \cdot (n-n_{r})).  \end{array} $$

By using the above recurrence formulae, *N*(*s*,*n*) is calculated for *s*∈{*s*_*m*_−*K*,*s*_*m*_−1} and $n \in \{n_{\min }, \dots, n_{\max }\}$, where *s*_*m*_ is the start position of the STR region. Since the repeat pattern size *u* is usually less than or equal to four and can be considered as a constant, the calculation of *N*(*s*,*n*) requires $O((n_{\max }-n_{\min }+1)K)$ time, which is smaller than that required in the naïve way.

### Repeat number estimation considering genealogy of multiple individuals

DNA sequences are inherited from parents to offspring, and single base substitutions occur in the inheritance with mutation rate of around 2.0×10^−8^ [16]. Repeat numbers in STR regions also change or mutate in the inheritance from a parent to its offspring with rate ranging usually from 1.0×10^−4^ to 1.0×10^−3^ [17]. From the phased genotypes around an STR region of interest for multiple individuals, we consider their genealogy around the region by using coalescent tree [11–13]. Coalescent tree is a binary tree in which leaves represent the current haplotypes and internal nodes represent past coalescent events of the haplotypes. For each coalescent event, two linages are involved, and cases involving more than two lineages are not considered in our model. The length of each edge in the tree represents time between coalescent events.

We propose a new statistical model named coalescentSTR that uses coalescent trees estimated from phased genotypes around an STR region to connect the basic models of multiple individuals for more accurate estimation of their repeat numbers. Repeat numbers in the STR region obey the estimated coalescent tree. Thus, given nearby phased genotypes *V*, we consider the prior distribution of repeat numbers via coalescent trees estimated from *V* and model insert size inferred from paired-end reads in the following formula. Let $l_{i}^{(d)}$ be an insert size of the *d*th read pair for the *i*th individual. We also denote $n_{i_{1}}$ and $n_{i_{2}}$ as repeat numbers of haplotype 1 and haplotype 2 for the *i*th individual, respectively. We represent the likelihood of insert size $l_{i}^{(d)}$ as

2 $$\begin{array}{@{}rcl@{}} {}P(L, N \! \mid\! V) \! =\! \prod\limits_{i=1}^{I}\! \prod\limits_{d=1}^{D_{i}} \! P(l_{i}^{(d)} \! \mid\! n_{i_{1}},n_{i_{2}})\! \! \sum\limits_{g}\! P(N \! \mid\! g) P(g \! \mid\! V), \end{array} $$

where *I* is the number of individuals, *L* is a set of $l_{i}^{(d)}$, *N* is a set of $n_{i_{1}}$ and $n_{i_{2}}$, *D*_*i*_ is the number of read pairs for individual *i*, and *g* is a coalescent tree. The first term in the right hand of Eq. (2) is given by the likelihood function of the basic model. In the second term, repeat numbers are connected by coalescent tree *g* as

3 $$ {}P(N\! \! \mid \!g) \! =\! \sum\limits_{n_{c_{1}}=n_{\min}}^{n_{\max}} \! \cdots \! \sum\limits_{n_{c_{I-1}}=n_{\min}}^{n_{\max}} \prod\limits_{v \in C_{g}} \prod\limits_{u \in o_{v|g}} \! P\left(n_{u} \! \mid\! n_{v}, t_{v,u|g}; \mu_{s}\right)\!,  $$

where *C*_*g*_ is a set of internal nodes $c_{1}, \dots, c_{I-1}$ in *g*, *o*_*v*|*g*_ is a set of offspring nodes of *v* in *g*, *t*_*v*,*u*|*g*_ is coalescent time from node *v* to *u* in *g*, and *n*_*v*_ is a repeat number in node *v*. Note that the size of *o*_*v*|*g*_ is two. *P*(*n*_*u*_∣*n*_*v*_,*t*_*v*,*u*|*g*_;*μ*_*s*_) represents the change of repeat numbers from parent node *v* to its offspring node *u* in time *t*_*v*,*u*|*g*_ with mutation rate *μ*_*s*_. For the change of repeat numbers, we consider the stepwise model [18], where repeat numbers change at most one in one generation with mutation rate *μ*_*s*_. With the Brownian motion approximation to the stepwise model [19], *P*(*n*_*u*_∣*n*_*v*_,*t*_*v*,*u*|*g*_;*μ*_*s*_) is given by

$$P(n_{u} \mid n_{v}, t_{v,u|g}; \mu_{s})\! = \!\min\{1, \mathcal{N}\left(n_{u}; n_{v}, N_{e} \cdot \mu_{s} \cdot t_{v,u|g}\right)\}, $$

where $\mathcal {N}$ represents the normal distribution and *N*_*e*_ is the effective population size. *P*(*g*∣*V*) represents the probability of coalescent tree *g* given nearby phased genotypes *V*. Since it is infeasible to calculate the summation in all possible coalescent trees *g* in Eq. (2), we sample a set of coalescent trees $\mathcal {G}$ from the phased genotypes *V* with MCMC [13, 20], and calculate the summation only on $g \in \mathcal {G}$:

4 $$\begin{array}{@{}rcl@{}} P(L, N \mid V) = \prod\limits_{i=1}^{I} \prod\limits_{d=1}^{D_{i}} P\left(l_{i}^{(d)} \mid n_{i_{1}},n_{i_{2}}\right) \sum\limits_{g \in \mathcal{G}} P(N \mid g). \end{array} $$

For sampling with MCMC, burn-in period, period between samples, and the number of samples are respectively set to 50,000, 100, and 100 in our study.

### Estimation of repeat numbers in coalescentSTR

In coalescentSTR, repeat numbers are estimated by finding *N* maximizing Eq. (4):

$$\hat{N}= \arg \max_{N} \prod\limits_{i=1}^{I} \prod\limits_{d=1}^{D_{i}} P\left(l_{i}^{(d)} \mid n_{i_{1}},n_{i_{2}}\right) \sum\limits_{g \in \mathcal{G}} P(N \mid g). $$

The calculation of the exact value of $\hat {N}$ is known as the marginal MAP problem and NP-hard even when the model structure including other hidden variables is a tree [15]. If the number of trees in $\mathcal {G}$ is one, i.e., the summation in *g* is not considered, the mixed-product belief propagation (mixed-product BP) [15] can be applied to obtain an approximated solution of $\hat {N}$. However, if the summation in *g* is considered, the mixed-product belief propagation cannot be applied directly, and a new algorithm is required for the solution. Here, we propose a new belief propagation algorithm named multiple-tree belief propagation (multiple-tree BP), which considers belief propagation in multiple trees. Given messages to variables in *N*∖{*n*_*i*1_}, we consider message passing from variables in *N*∖{*n*_*i*1_} to *n*_*i*1_. In multiple-tree BP, message to *n*_*i*1_ on each tree is calculated independently. Since message passing can be calculated exactly on tree structures by the belief propagation, a message to *n*_*i*1_ can be obtained by taking the summation of messages to *n*_*i*1_ from trees. Multiple-tree BP is extended to the loopy belief propagation (loopy BP) [14] and mixed-product BP. We first consider multiple-tree BP for loopy BP, and then describe its extension to mixed-product BP later. A message from internal node *v* in coalescent tree *g* to its parent *p*_*v*∣*g*_ is given by

$$ \begin{aligned} {}m_{v \rightarrow p_{v \mid g}} (n_{p_{v \mid g}})\,=\, \sum\limits_{n_{v}=n_{\min}}^{n_{\max}} \!P\left(n_{u} \!\mid\! n_{v}, t_{v,u|g}; \mu_{s}\right) \cdot m_{o_{1} \rightarrow v}(n_{v}) \!\cdot\! m_{o_{2} \rightarrow v}(n_{v}),  \end{aligned}  $$

where *o*_1_ and *o*_2_ are offspring of *v*. On the other hand, a message from a leaf node *i*_1_ to its parent $p_{i_{1} \mid g}$ in coalescent tree *g* is given by

5 $$ \begin{aligned} {}m_{i_{1} \rightarrow p_{i_{1} \mid g}} (n_{p_{i_{1} \mid g}}) = \sum\limits_{n_{i_{1}}=n_{\min}}^{n_{\max}} P(n_{i_{1}} \mid n_{p_{i_{1} \mid g}}, t_{p_{i_{1} \mid g}}; \mu_{s}) \cdot m_{i_{2} \rightarrow i_{1}}(n_{i_{1}}), \end{aligned}  $$

where $m_{i_{2} \rightarrow i_{1}}(n_{i_{1}})$ is a message from $n_{i_{2}}$ to $n_{i_{1}}$. A message from internal node *v* to one of its offspring *o*_1_ is given by

$$ \begin{aligned} {}m_{v \rightarrow o_{1}} (n_{o_{1}}) \,=\, \sum\limits_{n_{v}=n_{\min}}^{n_{\max}} \!\!P(n_{i_{1}} \!\!\mid \!n_{p_{i_{1} \mid g}}, t_{p_{i_{1} \mid g},i_{1}}; \mu_{s})\! \cdot\! m_{p_{v \mid g} \rightarrow v}(n_{v}) \!\cdot\! m_{o_{2} \rightarrow v}(n_{v}),  \end{aligned}  $$

where *o*_2_ is a sibling of *o*_1_. A message from $n_{i_{1}}$ to $n_{i_{2}}$ is calculated by

6 $$ \begin{aligned} {}m_{i_{1} \rightarrow i_{2}}(n_{i_{2}})\! =\! \sum\limits_{n_{i_{1}}=n_{\min}}^{n_{\max}} \prod\limits_{d=1}^{D_{i}} \!P(l_{i}^{(d)} \!\mid\! n_{i_{1}},n_{i_{2}}) \!\cdot\! \sum\limits_{g \in \mathcal{G}} \!m_{p_{i_{1} \mid g} \rightarrow i_{1}}(n_{i_{1}}). \end{aligned}  $$

**Fig. 1:**
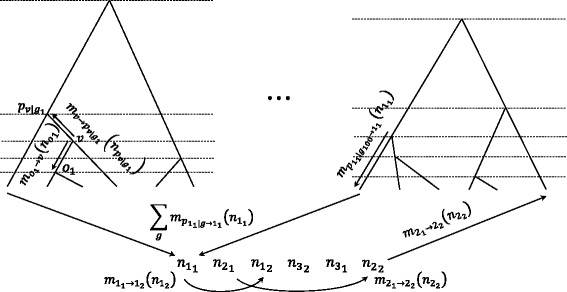
Coalescent trees on three individuals and messages in the belief propagation. The flow of messages from *v* to $p_{v \mid g_{1}}$, from *v* to its offspring *o*_1_, from leaf 1_1_ to leaf 1_2_, from leaf 2_1_ to leaf 2_2_, from $p_{1_{1} \mid g_{100}}$ to leaf 1_1_, and from trees to leaf 1_1_ are illustrated

Figure 1 illustrates the flow of the above messages. For the extension to mixed-product BP, the message from $n_{i_{1}}$ to $n_{i_{2}}$ in Eq. (6) is replaced with

$$\begin{aligned} {}m_{i_{1} \rightarrow i_{2}}(n_{i_{2}})\! = \max_{n_{i_{1}}} \left\{\prod\limits_{d=1}^{D_{i}} P(l_{i}^{(d)} \mid n_{i_{1}},n_{i_{2}}) \sum\limits_{g \in \mathcal{G}} m_{p_{i_{1} \mid g} \rightarrow i_{1}}(n_{i_{1}})\right\}\!, \end{aligned} $$

and Eq. (5) is replaced with

$$ \begin{aligned} {}\tilde{m}_{i_{1} \rightarrow p_{\left\{i_{1} \mid g\right\}}} \!\!\left(\!{n_p}_{\left\{i_{1} \mid g\right\}}\!\right) \,=\,\! \sum\limits_{n_{i_{1}} \in \boldsymbol{n}_{i_{1}}} \!\!P\!\left(\!n_{i_{1}},\!{n_p}_{\left\{i_{1} \mid g\right\}}; \mu_{s}, t_{p_{\left\{i_{1} \mid g\right\},}i_{1}}\!\right) \cdot m_{i_{2} \rightarrow i_{1}}(n_{i_{1}}),  \end{aligned}  $$

where $\boldsymbol {n}_{i_{1}}$ is a set of $n_{i_{1}}$ maximizing $m_{i_{2} \rightarrow i_{1}}(n_{i_{1}})\!\!\!\!\cdot \sum _{g \in \mathcal {G}} m_{p_{i_{1} \mid g} \rightarrow i_{1}}(n_{i_{1}})$. After some iterations of mixed-product BP, $\hat {n}_{i_{1}} \in \hat {N}$ is obtained by $\arg \max _{n_{i_{1}}} m_{i_{2} \rightarrow i_{1}}(n_{i_{1}}) \cdot \sum _{g \in \mathcal {G}} m_{p_{i_{1} \mid g} \rightarrow i_{1}}(n_{i_{1}})$. We first calculate loopy BP in ten cycles and then calculate mixed-product BP in ten cycles using messages from loopy BP as initial values. Empirically, the above procedure provides better $\hat {N}$ than only considering mixed-product BP.

### Selection of STR mutation rate

Messages from loopy BP is used to calculate the following value:

7 $$ \begin{aligned} {}\sum_{i=1}^{I} \!\left(\!\sum\limits_{n_{i_{1}}=n_{\min}}^{n_{\max}} \bar{\!m}_{i_{2} \rightarrow i_{1}}(n_{i_{1}}) \!\cdot\! \bar{m}_{\mathcal{G} \rightarrow i_{1}}\!(n_{i_{1}})\! +\! \sum\limits_{n_{i_{2}}=n_{\min}}^{n_{\max}} \bar{\!m}_{i_{1} \rightarrow i_{2}}(n_{i_{2}}) \!\cdot\! \bar{m}_{\mathcal{G} \rightarrow i_{2}}(n_{i_{2}})\!\right)\!, \end{aligned}  $$

where $\bar {m}_{i_{2} \rightarrow i_{1}}(n_{i_{1}})$ is $m_{i_{2} \rightarrow i_{1}}(n_{i_{1}})$ normalized to have a sum of one and $\bar {m}_{\mathcal {G} \rightarrow i_{1}}(n_{i_{1}})$ is a message to $n_{i_{1}}$ from coalescent trees $\sum _{g \in \mathcal {G}} m_{p_{i_{1} \mid g}}(n_{i_{1}})$ normalized to have a sum of one. We consider that messages from paired-end reads to $n_{i_{1}}$ or $n_{i_{2}}$ and from coalescent trees to $n_{i_{1}}$ or $n_{i_{2}}$ are similar to each other if the STR mutation rate is proper. The value in Eq. (7) is designed to take higher value if those messages are more similar to each other. We consider several STR mutation rates and select the rate with the highest value given by Eq. (7).

## Results and discussion

### Simulation analysis

Given a target STR region, we first synthetically generated repeat numbers of the STR region and nearby phased genotypes for 2*I* haplotypes as follows:

- Generate a coalescent tree for 2*I* haplotypes with an algorithm in [18] under the assumption of a constant effective population size.
- Obtain phased genotypes at 1,000 bp upstream and downstream positions of the STR region based on the generated coalescent tree and a specified single base substitution rates.
- Obtain repeat numbers based on the generated coalescent tree by considering the stepwise model with a specified STR mutation rate.

The effective population size was set to 10,400 [21], and single base substitution rates on transition and transversion were set to 5.5×10^−8^ and 1.2×10^−8^, respectively as in [16]. For the STR region, we considered TTTC repeat region at chr7:127898719-127898787 in the human reference genome (GRCh37) from tandem repeat regions detected by Tandem Repeats Finder [22]. The reference repeat number, the repeat number in the reference genome for the region, is 17. We obtained synthetically generated diploid genome sequences for each individual by editing GRCh37 chromosome 7 sequence according to repeat numbers in the region and phased genotypes around the region generated by the above procedures. The following settings were considered for the number of individuals and mutation rate:

- Five types of the numbers of individuals: 5, 10, 20, 50, and 100.
- Two types of STR mutation rates: 1.00×10^−3^ and 2.73×10^−4^. The former rate is an estimated STR mutation rate for tetranucleotide repeats, and the latter for dinucleotide repeats in human [17].

From each edited diploid genome pair, paired-end read reads with length of 100 bp and 0.1 % base substitution errors were generated in FASTQ format. Insert size of each read pair is normally distributed with mean 350 bp and standard deviation 50 bp. For the read coverage for each individual, 20 × and 40 × were considered. The generated pair-end read data was aligned to the reference genome with BWA-MEM [23]. We set the repeat number on the root of the generated coalescent trees to 25 to obtain repeat numbers with the size close to the read length. For coalescentSTR and the basic model, read pairs satisfying the following conditions were extracted for obtaining the insert size data for each individual:

- Directions of paired-end reads are concordant.
- The start position of the forward read in each aligned read pair is located before the start position of the STR region.
- The end position of the reverse read in each aligned read pair is located after the end position of the STR region.

Let $n_{i_{1}}$ and $n_{i_{2}}$ be true repeat numbers for the *i*th individual. We also let $\hat {n}_{i_{1}}$ and $\hat {n}_{i_{2}}$ be estimated repeat numbers for the *i*th individual. For the evaluation, we considered a root mean squared error (RMSE) between true and estimated repeat numbers given by

$$\begin{aligned} {}\sqrt{\frac{1}{2I} \sum\limits_{i=1}^{I} \min\{(n_{i_{1}}-\hat{n}_{i_{1}})^{2}+(n_{i_{2}}-\hat{n}_{i_{2}})^{2},(n_{i_{1}}-\hat{n}_{i_{2}})^{2}+(n_{i_{2}}\!-\hat{n}_{i_{1}})^{2}\}}. \end{aligned} $$

We evaluated the performance of coalescentSTR, the basic model, lobSTR [8], RepeatSeq [9], and STRViper [10]. In coalescentSTR, an STR mutation rate was selected from rates in {0.01,0.1,0.5,0.75,1,1.2,2,5,10,100} multiplied by the true STR mutation rate based on the value given in Eq. (7). $n_{\max }$ and $n_{\min }$ were set to 40 and zero, respectively. For each condition, we prepared ten coalescent trees and generated sequence datasets from them. In order to examine the effect of considering the genealogy, we randomly shuffled haplotypes on phased genotypes and used them for coalescentSTR. Tables 1 and 2 show RMSE values for results from coalescentSTR, coalescentSTR with the shuffled haplotypes (coalescentSTR shuffled), the basic model, lobSTR, RepeatSeq, and STRViper averaged on ten trials for the five types of individual counts, STR mutation rates of 2.73×10^−4^ and 1.00×10^−3^, and read coverages of 20 × and 40 ×, respectively.

**Table 1:** Comparison of estimated repeat numbers in terms of RMSE for simulation datasets with STR mutation rates of 2.73×10^−4^ and 1.00×10^−3^ and read coverage of 20 ×

STR mutation rate	2.73×10^−4^					1.00×10^−3^				
No. of samples	5	10	20	50	100	5	10	20	50	100
CoalescentSTR	**2.30**	2.46	**2.17**	**1.25**	**1.09**	4.09	**3.22**	**2.24**	**1.96**	**1.81**
CoalescentSTR (shuffled)	2.37	**2.42**	2.30	1.38	1.96	**4.02**	3.52	3.05	2.73	3.33
Basic Model	4.18	5.37	5.00	5.15	4.91	5.39	5.11	5.41	4.94	5.03
lobSTR	9.09	7.33	6.02	7.47	5.94	10.1	7.04	5.13	6.67	6.20
RepeatSeq	9.09	7.33	6.02	7.47	5.91	10.1	7.03	5.09	6.66	6.18
STRViper	8.38	6.87	5.57	6.91	5.60	9.37	6.59	4.90	6.23	5.75

Repeat numbers were estimated with datasets with 5, 10, 20, 50, and 100 individuals. The best result in each condition is in bold

**Table 2:** Comparison of estimated repeat numbers in terms of RMSE for simulation datasets with STR mutation rates of 2.73×10^−4^ and 1.00×10^−3^ and read coverage of 40 ×

STR mutation rate	2.73×10^−4^					1.00×10^−3^				
No. of samples	5	10	20	50	100	5	10	20	50	100
CoalescentSTR	**2.47**	**2.20**	**1.86**	**1.11**	**1.00**	**3.28**	**2.75**	**2.18**	**1.74**	**1.61**
CoalescentSTR (shuffled)	2.58	2.32	2.16	1.38	1.94	3.66	3.36	2.91	2.73	3.03
Basic Model	4.18	4.48	4.39	4.18	4.07	4.74	4.09	4.33	4.17	4.19
lobSTR	9.09	7.32	6.02	7.47	5.94	10.10	7.05	5.12	6.67	6.20
RepeatSeq	9.10	7.34	6.02	7.47	5.94	10.08	7.03	5.11	6.65	6.18
STRViper	7.93	6.55	5.32	6.57	5.37	8.88	6.32	4.81	5.97	5.51

Repeat numbers were estimated with datasets with 5, 10, 20, 50, and 100 individuals. The best result in each condition is in bold

If no STR variant was detected, the reference repeat number, 17, was assigned as the estimated repeat number. STRViper reports only one repeat number for each individuals although each individual has two repeat numbers. Thus, two repeat numbers in each individual were set to the same value in results of STRViper. CoalescentSTR gives the best result in most of the conditions and coalescentSTR (shuffled) gives the best result in some conditions with sample sizes of 5 and 10. If the sample size considered for estimation is small, the improvement of the performance by considering multiple individuals in coalescentSTR is limited. Thus, coalescentSTR (shuffled) can provide better results than coalescentSTR for some conditions with small sample sizes.

Since some repeat numbers are longer than or equal to the read length, the results from paired-end read distance based methods (coalescentSTR, basic model, and STRViper) are better than those from methods counting repeat numbers in sequences reads (lobSTR and RepeatSeq). The RMSE value is smaller for considering more individuals on coalescentSTR. In addition, the performance of coalescentSTR with the shuffled haplotypes is worse than that of coalescentSTR with correct haplotypes. These observations support the effectiveness of considering the genealogy. The RMSE value for each method on the dataset with read coverage of 40 × is smaller than that on the dataset with read coverage of 20 × in most of the cases.

**Fig. 2:**
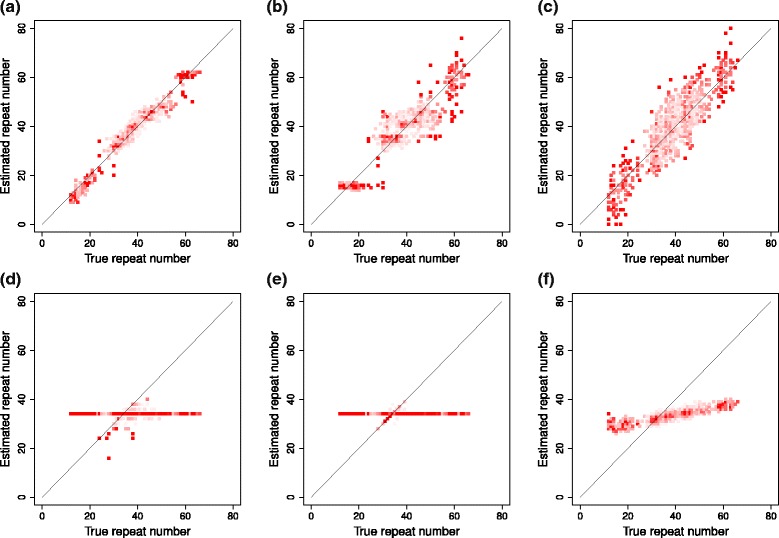
A plot comparing sums of true repeat numbers and estimated repeat numbers for each individual on simulation data with mutation rate of 1.00×10^−3^ and read coverage of 40 ×. The x-axis and y-axis indicate the sum of true diploid repeat numbers and the sum of estimated diploid repeat numbers for one individual, respectively. Plots for coalescentSTR, coalescentSTR (shuffled), Basic Model, lobSTR, RepeatSeq, and STRViper are receptively in (**a**), (**b**), (**c**), (**d**), (**e**), and (**f**)

Figure 2 (a), (b), (c), (d), (e), and (f) show plots for comparing the sum of estimated diploid repeat numbers for one individual and the sum of corresponding true diploid repeat numbers for coalescentSTR, coalescentSTR (shuffled), Basic Model, lobSTR, RepeatSeq, and STRViper, respectively. The simulation datasets with STR mutation rate of 1.00×10^−3^ and read coverage of 40 × are used in the plots. In each plot, the x-axis indicates the sum of true diploid repeat numbers for one individual and the y-axis indicates the sum of estimated diploid repeat numbers. Ideally, points in plots are located on the diagonal line.

In the plot for coalescentSTR, points are around the diagonal line. Points in the plot for coalescentSTR (shuffled) are also located around the diagonal line, but scattered in larger area than those in the plot for coalescentSTR. In addition, points in the plot for the basic model are scattered in larger area around the diagonal line than those in plots for coalescentSTR and coalescentSTR (shuffled). There exists a horizontal line with the value twice as much as the reference repeat number in plots for lobSTR and RepeatSeq The line is due to points for cases where these methods failed to STR variants and provided the reference repeat number as estimated repeat numbers. For cases with STR variants which can be detected by RepeatSeq, the corresponding points are located around the diagonal line tightly. On the other hand, points associated with STR variants which can be detected by lobSTR are scattered around the diagonal line. In the plot for STRViper, the sum of estimated diploid repeat numbers is correlated with the sum of true diploid repeat numbers, but differences between estimated repeat numbers and the reference repeat number are underestimated.

### Real data analysis

We evaluated the performance of coalescentSTR, basic model, lobSTR, RepeatSeq, and STRViper using exome sequencing data of JPT individuals and WGS data of CEU and GBR individuals.

#### Performance evaluation with exome sequencing data

**Fig. 3:**
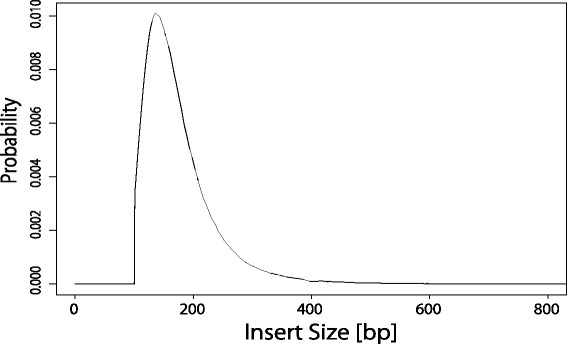
An empirically obtained insert size distribution. This empirical distribution was generated from aligned read pairs in exome data of a JPT individual in 1KGP sequenced with Illumina HiSeq

We evaluated the performance of coalescentSTR, basic model, lobSTR, RepeatSeq, and STRViper on an STR region comprised of TCA repeats located in the exon region of CENPP at chr9:95237025-95237069 by using 1KGP exome datasets for 33 HapMap JPT individuals [5]. The reference repeat number is 14, and repeat numbers in the region range mainly from 11 to 16. Read length of datasets for some individuals is 100 bp and that for remaining individuals is 75 bp. Since the total length of the STR region is included in the read length, repeat numbers can be inferred directly from the sequence reads spanning the region. In order to evaluate performance on repeats with the size close to the read length, we truncated the tail of each read to obtain paired-end sequence datasets with length of 50 bp in FASTQ format. For the true repeat numbers of the datasets, repeat numbers estimated from the original datasets with lobSTR were used. Sequence reads in the obtained datasets were aligned to the reference genome with BWA-MEM. Insert size distribution was obtained from the datasets for each individual. Phased genotypes around the STR region for coalescentSTR were obtained from the 1KGP Phase3 imputation panels released in October, 12, 2014 [5]. Phased genotypes at 4,000 bp upstream and downstream positions of the STR region were used for sampling coalescent trees. An STR mutation rate was selected from {0.0001,0.0005,0.001,0.005,0.01} based on the value given by Eq. (7). $n_{\max }$ and $n_{\min }$ were set to 40 and zero, respectively. RMSE values in the results from coalescentSTR, coalescentSTR (shuffled), the basic model, lobSTR, RepeatSeq, and STRViper on the datasets with read length of 50 bp are summarized in Table 3. Since read length of 50 bp is not sufficient for the detecting the repeat patterns directly from sequence reads including the STR region, no STR variant was detected in lobSTR and RepeatSeq for all the individuals. Although STRViper assumes the normality on insert size distribution, the actual insert size in the datasets is not normally distributed as shown in Fig. 3, and hence STRViper may fail to detect the STR variants. For cases of detecting no STR variant, the reference repeat number was assigned for the estimated repeat numbers. CoalescentSTR gives the best RMSE, and the basic model gives the worst RMSE. Since the quality of the data is different between datasets, the basic model failed to estimate repeat numbers for low quality datasets. In addition, since the true repeat numbers do not vary a lot, the results with the reference repeat number for all the individuals give not so bad RMSE, and hence the result of the basic model is the worst among the methods.

**Table 3:** Comparison of estimated repeat numbers in terms of RMSE for real exome data for JPT individuals in 1KGP

Method	CoalescentSTR	CoalescentSTR	Basic model	lobSTR	RepeatSeq	STRViper
		(shuffled)				
RMSE	**1.33**	2.38	9.44	1.63	1.63	1.63

The best result is in bold

**Fig. 4:**
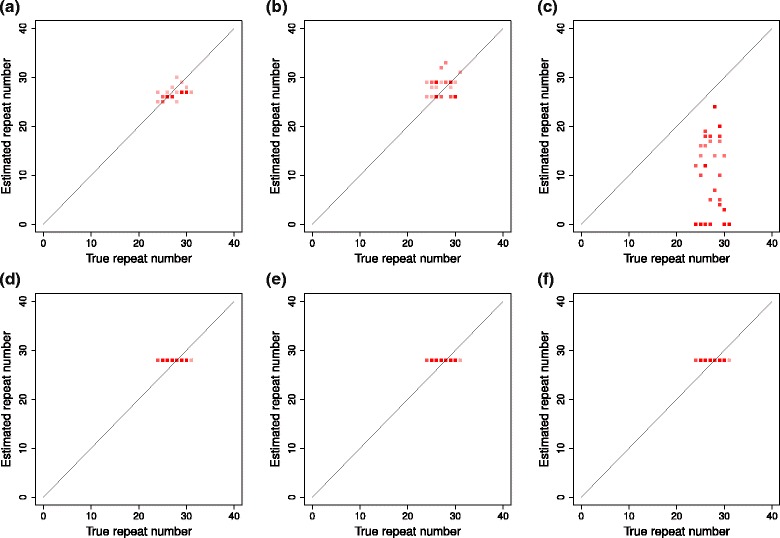
A plot comparing sums of true repeat numbers and estimated repeat numbers for each individual on real data. The x-axis and y-axis indicate the sum of true diploid repeat numbers and the sum of estimated diploid repeat numbers for one individual, respectively. Plots for coalescentSTR, coalescentSTR (shuffled), Basic Model, lobSTR, RepeatSeq, and STRViper are receptively in (**a**), (**b**), (**c**), (**d**), (**e**), and (**f**)

Figure 4 (a), (b), (c), (d), (e), and (f) show plots for comparing the sum of estimated diploid repeat numbers for one individual and the sum of corresponding true diploid repeat numbers for coalescentSTR, coalescentSTR (shuffled), the basic model, lobSTR, RepeatSeq, and STRViper for real data set, respectively.

In the plot for coalescentSTR, points are located around the diagonal line. Points in the plot for coalescentSTR (shuffled) are also located around the diagonal line, but scattered in larger area than those in the plot for coalescentSTR. In the plot for the basic model, the sum of estimated repeat numbers is not correlated with the sum of true repeat numbers because the amount data for each individual in this experiment is not sufficient for estimating repeat numbers correctly. Since lobSTR, RepeatSeq, and STRViper could not detect STR variants for any sample, only a horizontal line with the value twice as much as the reference repeat number is observed in the plots for lobSTR, RepeatSeq, and STRViper.

#### Performance evaluation with WGS data

We applied coalescentSTR and other existing methods to WGS data of a HapMap CEU individual, NA12878 from HiSeq 2000 to estimate numbers of CAC repeats at chr1:20200573-20200666 in GRCh37 for NA12878. Read length and average insert size of the WGS data are respectively 101 bp and 300 bp, and its read coverage is 50 ×. The data was provided by the Illumina Platinum Genomes Project through the European Nucleotide Archive under the study accession PRJEB3381 (http://www.ebi.ac.uk/ena/data/view/ERP001960). In addition to the WGS data of NA12878, we used WGS data for 35 HapMap CEU and 35 HapMap GBR individuals released in May, 22, 2012 by 1KGP [5]. Read length and read coverage of the WGS data of these 70 individual is 100 bp and 5 ×, respectively. Sequence reads in the WGS data of NA12878 were aligned with BWA-MEM while those in the WGS of others were aligned with BWA [24]. Phased genotypes around the STR region for coalescentSTR were obtained from the 1KGP Phase3 imputation panels released in October, 12, 2014 [5]. For sampling coalescent trees, phased genotypes at 3,000 bp upstream and downstream positions of the STR region were used. An STR mutation rate was selected from {0.0001,0.0005,0.001,0.005,0.01} based on the value given by Eq. (7). $n_{\max }$ and $n_{\min }$ were set to 40 and zero, respectively.

The size of the STR region is 93 bp in the reference genome, and it is difficult to estimate repeat numbers in the region by directly counting repeat numbers in the aligned reads for the data of read length of 100 bp due to the lack of aligned bases to the flanking regions in spanning reads. Thus, we estimated repeat numbers estimated from high coverage sequencing data with long reads, and used the estimated repeat numbers as true repeat numbers for the evaluation. For sequencing data with long reads, PacBio sequencing data for NA12878 provided from GIAB Reference Materials and Data (ftp://ftp-trace.ncbi.nlm.nih.gov/giab/ftp/data/NA12878/NA12878_PacBio_MtSinai) [25] was used, and repeat numbers were estimated by the following procedures:

- Error-corrected reads with Falcon (https://github.com/PacificBiosciences/FALCON) in FASTA format were aligned to GRCh37 with BWA-MEM.
- The number of bases aligned in the STR region was counted for each read spanning the region.
- Two-component Gaussian mixture model was applied to the set of numbers of bases obtained the above, and estimated means for two components divided by the size of the repeat pattern were adopted as estimated repeat numbers.

The estimated repeat numbers from the above procedures were 28.02 and 25.38 and used for the evaluation by calculating RMSE given as follows:

$$\begin{aligned} {}\sqrt{\frac{1}{2} \min\{(28.02-\hat{n}_{1})^{2}\,+\,(25.38\,-\,\hat{n}_{2})^{2},(28.02-\hat{n}_{2})^{2}\,+\,(25.38-\hat{n}_{1})^{2}\}}, \end{aligned} $$

where $\hat {n}_{1}$ and $\hat {n}_{2}$ are estimated repeat numbers for NA12878. The estimated repeat numbers from coalescentSTR, coalescentSTR (shuffled), the basic model, lobSTR, RepeatSeq, and STRViper and their corresponding RMSE values with the repeat numbers estimated from the PacBio data are summarized in Table 4. Since no variant was detected by lobSTR and RepeatSeq, the reference repeat number, 31, was assigned to their estimated results. Similarly to the results in the former real data experiment, coalescentSTR gives the best RMSE and the basic model gives the worst RMSE.

**Table 4:** Estimated repeat numbers from WGS data from HiSeq 2000 for NA12878 and their corresponding RMSE values with the repeat numbers estimated from PacBio sequencing data for NA12878

Method	Estimated repeat numbers	RMSE
CoalescentSTR	28/26	**0.44**
CoalescentSTR (shuffled)	25/25	2.15
Basic Model	33/32	5.46
lobSTR	31/31	4.50
RepeatSeq	31/31	4.50
STRViper	31.12/31.12	4.61

The best result is in bold

### Comparison of computational time

Table 5 shows the computational time of coalescentSTR, the basic model, lobSTR, RepeatSeq, and STRViper for the simulation data in Section Simulation analysis and the real data for HapMap JPT individuals in Section Performance evaluation with exome sequencing data. For simulation data, the dataset with 100 individuals and STR mutation rate of 2.73×10^−4^ is considered. Note that read alignment time with lobSTR is not included for the computational time of lobSTR because read alignment time with BWA-MEM is not included in those of other algorithms. All the computation was performed on Intel Xeon CPU E5-2670 processors with single thread. CoalescentSTR and the basic model are implemented in Java. For coalescentSTR, computation time for sampling coalescent trees and estimation using sampled trees are separated in Table 5 as coalescentSTR (sampling) and coalescentSTR (estimation). In both simulation and real data, coalescentSTR requires the most computational time, especially in sampling coalescent trees. Computational time required for estimation by coalescentSTR is slightly more than that of STRViper. For memory consumption, coalescentSTR requires less than 4GB in both sampling and estimation.

**Table 5:** Comparison of computational time on the simulation dataset with 100 individuals, STR mutation rate of 2.73 × 10^4^, and read coverage of 40 × and the real dataset for HapMap JPT individuals

Method	Computational time	Computational time
	(simulation data)	(real data)
CoalescentSTR (sampling)	13372.96 [s]	846.60 [s]
CoalescentSTR (estimation)	452.59 [s]	66.86 [s]
Basic Model	49.58 [s]	16.18 [s]
lobSTR	2.72 [s]	8.17 [s]
RepeatSeq	10.24 [s]	14.78 [s]
STRViper	407.03 [s]	83.45 [s]

## Conclusions

We proposed a statistical approach named coalescentSTR to estimate repeat numbers in an STR region for multiple individuals from insert size data obtained by paired-end reads in HTS data. We considered the genealogy of the multiple individuals and used the genealogy for propagating repeat number information from insert size among individuals to achieve more accurate estimation of repeat numbers. We evaluated the performance of coalescentSTR, the basic model, lobSTR, RepeatSeq, and STRViper from simulation data and real data from 1KGP and verified the effectiveness of coalescentSTR for STR regions longer than or equal to the read length.

For computational time, coalescentSTR requires the most computational time from the comparison with other existing methods, and its computational time is mainly taken by sampling coalescent trees with MCMC. The use of MCMC with approximate Bayesian computation (ABC) [26] is a solution addressing this issue because the calculation of likelihood for each sampled tree is avoided with ABC and the calculation mainly requires the computational time for sampling. For larger size of genome structural variations such as large size copy number variations, the recombination of genomes needs to be considered although the recombination is basically not considered in coalescent theory. We are considering to extend the proposed model in future work in order to use ancestral recombination graph, which can handle the recombination in the genealogy of multiple individuals unlike coalescent tree.
